# Young Patients With Persistent and Complex Care Needs Require an Integrated Care Approach: Baseline Findings From the Multicenter Youth Flexible ACT Study

**DOI:** 10.3389/fpsyt.2020.609120

**Published:** 2020-11-25

**Authors:** Marieke Broersen, Nynke Frieswijk, Hans Kroon, Ad A. Vermulst, Daan H. M. Creemers

**Affiliations:** ^1^GGZ Oost Brabant, Oss, Netherlands; ^2^Tranzo – Tilburg School of Social and Behavioral Sciences, Tilburg University, Tilburg, Netherlands; ^3^Accare, Groningen, Netherlands; ^4^Trimbos Institute, Utrecht, Netherlands

**Keywords:** mental health services, adolescents, complex care needs, assertive community treatment, integrated care approach, fragmented care, intensive case management

## Abstract

**Background:** The Multicenter Youth Flexible ACT Study is an ongoing observational prospective cohort study that examines the effects of Youth Flexible ACT (Assertive Community Treatment) on young people with complex care needs who are difficult to engage in traditional (office-based) mental health services. However, a clear and detailed description of this patient group is lacking. In the current paper, we present baseline characteristics and psychosocial outcomes of the Youth Flexible ACT target group and explore the existence of underlying specific patient subgroups.

**Methods:** Sixteen Youth Flexible ACT teams from seven mental healthcare institutes in the Netherlands participated in the study. Research participants were monitored for 18 months and administered questionnaires measuring psychiatric- and social functioning every 6 months, yielding four measurements. Baseline data were obtained from 199 adolescents, their mental health workers, and parents/carers. Latent Class Analysis based on HoNOSCA scores (measuring psychosocial and daily functioning) was conducted to identify underlying subgroups.

**Results:** The target group of Youth Flexible ACT mainly consisted of patients older than 15 years of age with a history of (specialized) mental healthcare. They face many complex problems, including trauma; developmental, mood, and anxiety disorders; and problems with school attendance, family life, and peer relationships. Other frequently reported difficulties were substance misuse, the involvement of the legal system or police, problems with intellectual functioning, and personal finance. Patients were classified into four distinct classes: the “internalizing,” “externalizing,” “non-specific,” and the “overly impulsive” subgroup. Each subgroup had its unique pattern of difficulties and focus, respectively, high levels of depression and anxiety, disruptive behavior, unspecific difficulties, and substance misuse.

**Conclusions:** As expected, patients in Youth Flexible ACT experienced many severe problems, rendering them vulnerable to fragmented and, thus, ineffective care. Our findings underscore the need for an integrated care approach with a multidisciplinary team of skilled professionals that can bridge these wide-ranging psychosocial problems, as each class of participants experienced a different set of difficulties. Youth Flexible ACT teams need to adjust their care services accordingly.

## Introduction

It is estimated that about 5% of Dutch children and adolescents have mental illnesses leading to functional impairment and hindered development (1, 2). For the majority, these problems are often temporary. For a small subgroup, however, these problems can be rather severe and persistent (1–5). Adolescents in need of longer-term mental healthcare usually face complex psychiatric and social problems in everyday life, including difficulties with education, employment, peer relationships, family, housing, finances, health, substance abuse, and the criminal justice system (2, 6–10). These young people are often raised in families unable to provide proper education and/or have family members who suffer from psychiatric, financial, or addiction problems (6, 7, 11, 12). Multiple life challenges and harsh living conditions affect their psychiatric difficulties and vice versa (8).

Unfortunately, appropriate and accessible care for adolescents with complex psychiatric and social problems is sparse. The well-known traditional outpatient (office-based) care and psychiatric inpatient care offer limited services to this specific patient group (13–16). Limited access and commitment to care increase drop-out (17–20). Reasons for treatment disengagement include the lack of stability in daily life, difficulty trusting services, growing tired of services, treatment discontinuity, and a fragmented healthcare system (11, 17–25). When adolescents face complex care needs, they often need multiple mental health and social services for their various problems. To navigate these various services requires an active role and a fairly high level of knowledge of the healthcare system. This is often too challenging for these adolescents and their parents, resulting in fragmented and discontinued care. Integrated care approaches are needed to bridge these wide-ranging problems that these adolescents and their families face and provide alternative options to psychiatric inpatient and traditional office-based care.

In the Netherlands, Youth Flexible Assertive Community Treatment (Flexible ACT) teams were set up to meet the needs of children and adolescents with complex care problems and to tackle the problems of fragmented care services. Youth Flexible ACT is the youth variant (0–24 years of age) of Flexible ACT for adults. It is the Dutch adaptation and elaboration of Assertive Community Treatment (ACT), which originated in the United States in the 1970s (26). Flexible ACT is a client-centered service delivery model in which integrated teams provide long-term assertive outreach care consisting of both treatment for psychiatric symptoms and practical assistance with daily living needs, rehabilitation, and recovery support. Youth Flexible ACT is particularly focused on collaboration with adolescents, families, and their (in)formal networks. This results in shared goals aimed at improving the functioning in multiple life domains, improving patients' participation in the community, and enhancing their quality of life (7, 9). A multidisciplinary team of professionals delivers a complete range of services on a continuum of care. Nowadays, ~60 teams are active or under development in the Netherlands (27). A detailed portrayal of Youth Flexible ACT is outlined in the Youth Flexible ACT model description (7) and our study protocol (28).

In our ongoing Multicenter Youth Flexible ACT Study (28), we examine the effect of the care model by investigating (1) improvement in treatment outcomes throughout the Flexible ACT care and (2) associations between (elements of) Youth Flexible ACT model fidelity and treatment outcomes. Theoretically, Youth Flexible ACT targets a specific patient group with specific care needs. As the first step in this line of research, this study investigates whether characteristics of patients in our study population match with the intended target population as specified by the model description (7). Thus, the current paper provides a detailed description of adolescents in 16 Youth Flexible ACT teams in the Netherlands. Investigating the Youth Flexible ACT target group will contribute to organizing, adjusting, and developing appropriate care for these adolescents. Specifically, the aim of this paper is to characterize the composition of the study population. We describe baseline characteristics and psychosocial outcomes of the overall population of 199 participants and explore potential patient subgroups.

## Materials and Methods

### Design

The Multicenter Youth Flexible ACT Study is an ongoing observational prospective cohort study (2017–2021) of 16 Youth Flexible ACT teams from 7 mental healthcare institutes in the Netherlands. Patients were monitored for 18 months and administered questionnaires every 6 months, yielding 4 measurements (T0, T1, T2, T3). In this current paper we described cross-sectional data from the baseline measurement (T0).

### Participants

According to the Youth Flexible ACT model description (7) young people <24 years of age eligible for Youth Flexible ACT if they adhere to the following criteria. They (1) are diagnosed with a mental health disorder (or presumptive diagnosis); (2) experience difficulties in multiple areas of daily life (for example, problems with education, employment, peer relationships, housing, personal finance, health, substance abuse, and issues with the criminal justice system); (3) face family system problems and/or parenting issues; (4) have difficulty accessing and remaining in traditional outpatient care or the traditional care proves to be unfruitful; and (5) live in the district of the Youth Flexible ACT team. Additionally, the following research inclusion criteria were used: patients had to be between 12 and 24 years of age, have sufficient knowledge of the Dutch language, and provide written informed consent (along with parent/caregivers' consent).

### Setting and Data Collection

The 16 Youth Flexible ACT teams are located in both the city and the rural areas in the Netherlands and are working according to the Youth Flexible ACT model guidelines (7). An official audit was performed by the Centre for Certification ACT and Flexible ACT (CCAF) to determine the degree to which each team complies with the Youth Flexible ACT model. Fidelity scores showed that all teams had implemented the model successfully. We will describe these model fidelity scores more throroughly in our future studies.

Team members asked eligible adolescents to participate in the study during the intake process. Participants, their parents/carers, and the mental healthcare worker were asked to complete a baseline measurement consisting of questionnaires within 12 weeks after signing informed consent. The baseline measurement was completed during a regular appointment by a familiar mental health worker or by patients independently in their own time. Both paper and online versions were available, although online versions were preferred to minimize missing data. Adolescent participants received a remuneration of €10. An online data system was used to collect the data. Confidentiality of the data was guaranteed through a two-factor authentication login procedure. Each institution had a unique digital environment. Before conducting baseline assessments, mental health workers were trained in administering the questionnaires. In particular, mental health workers received a HoNOSCA training based on the official HoNOSCA training (29, 30).

### Study Outcome Measures

The overall baseline characteristics and psychosocial outcomes were collected using questionnaires (Table 1) that assess general psychological functioning, specific diagnostic characteristics, and daily functioning of the participants. The employed set of questionnaires together reflect the multiple life domains in which Youth Flexible ACT operates. The questionnaires and the calculations of scores are described in detail in our study protocol (28). Additionally, sociodemographic and clinical characteristics (age, sex, clinical diagnoses) were collected from electronic patient records and via a sociodemographic questionnaire completed by mental health workers. To check whether research participants were representative of the Youth Flexible ACT population, age, sex and diagnoses of patients registered with the same Youth Flexible ACT teams during the same inclusion period were also collected. Furthermore, we conducted Latent Class Analysis (LCA) to identify the existence of specific patient subgroups based on their psychosocial- and daily functioning, as measured with the HoNSOCA (Health of the National Outcome Scales for Children and Adolescents) (30). We selected 10 out of the 13 HoNOSCA items that display the multiple life domains addressed by Youth Flexible ACT.

**Table 1:** Overview of outcome assessments at baseline.

**Variable**	**Instrument**
**Clinician-reported outcomes**	
Daily functioning	HoNOSCA
Sociodemographics	Sociodemographic questions
**Patient-reported outcomes**	
Psychosocial well-being	SDQ
Health-related quality of life	Kidscreen-10 + additional questions
Depressive symptoms	CDI-2
Social support	Scale “social support and peers” from the Kidscreen-52
Empowerment	Subscale “interactional empowerment” from the questionnaire EMPO 3.1
Psychosis risk screening	PQ-16
Treatment satisfaction	Four brief questions based on the Jeugdthermometer (Youth thermometer)
Care utilization	One question concerning care utilization
**Parent-reported outcomes**	
Psychosocial well-being–child	SDQ-P
Health-related quality of life–child	Kidscreen-10 parent version
Psychological distress	MHI-5
Parenting stress	PSQ-S
Treatment satisfaction	Four questions based on the Jeugdthermometer (Youth thermometer) parent version

*HoNOSCA, Health of the National Outcome Scales for Children and Adolescents; SDQ, The Strengths and Difficulties Questionnaire; CDI-2, Child Depression Inventory; EMPO 3.1, Empowerment questionnaire; PQ-16, The Prodromal Questionnaire; SDQ-P, The Strengths and Difficulties Questionnaire–parent version; MHI-5, Mental Health Inventory; PSQ-S, Parenting Stress Questionnaire–short form*.

### Participant Response Rates

During a 20-month enrolment period (October 2016–June 2018), 199 eligible patients, hereafter referred to as the monitoring sample, gave informed consent to participate, and completed baseline questionnaires on average 2.6 months after the start of Youth Flexible ACT care (SD = 2.38; range 0–14). Almost three quarters of the patients (74.9%) completed the baseline assessment within 16 weeks after starting with Youth Flexible ACT. In addition, age, sex, and diagnoses were obtained from 1,034 patients registered with the same Youth Flexible ACT teams during the same inclusion period, hereafter referred to as the non-monitoring sample. Demographic variables showed no significant differences between the monitoring and non-monitoring samples in terms of age (F_(1, 1231)_ = 0.99, *p* = 0.32) and sex (χ^2^ (1) = 1.82, *p* = 0.18). Regarding clinical diagnoses, only anxiety disorders were more prevalent in the monitoring sample than in the non-monitoring sample (24.6 vs. 15.6%, respectively, χ^2^ (1) = 9.27, *p* = 0.002, φ = −0.09). Yet, as the effect size was small, this difference was not substantial. This is an indication that the monitoring sample reflects the wider Youth Flexible ACT population in the Netherlands.

### Data Analysis

LCA was conducted to explore the patient composition, as measures with the HoNOSCA, using the statistical package Mplus version 7.2 (31). Missing values were 0 (4 items), 1 (4 items), 2 (1 item), and 8 (1 item). The Full Information Maximum Likelihood (FIML)-estimator was used as a default procedure to handle missing values. With this procedure, all available information in the data was used to estimate latent classes. Furthermore, patients were clustered within the 16 teams. Therefore, we used the TYPE = COMPLEX procedure in Mplus to correct parameter estimates for the dependency of patients within teams. Data management, descriptive statistics, and other statistical analyses were performed using SPSS version 25 (32).

#### Latent Class Analysis With Mplus

The 10 selected items of the HoNOSCA were used as indicators to classify patients into distinct subgroups (33). The responses to these items were dichotomized into “no problem/no action required” (scores 0 and 1) and “mild or severe problem/action required” (scores 2, 3, and 4) to facilitate model estimation (29). The starting moment of LCA is a 1-class solution followed by 2−, 3−, …, *k*+1 class solutions. Four criteria were used to identify the number of *k* classes. The first criterion is based on one or more information-theoretic criteria, like the Bayesian Information Criterion (BIC) (34), Akaike Information Criterion (AIC) (35), or the Sample Size Adjusted BIC (SSABIC) (36). We used the SSABIC as a metric for model performance, as it was shown to outperform both the BIC and AIC for small sample sizes (*N* < 200) (37). Lower SSABIC-values indicate improvement of model fit. If SSABIC-values for a model with *k*+1 classes increase, then a model with *k* classes is optimal. The second criterion is based on the classification quality of a model. High posterior probabilities indicate how well individuals are classified into their class, and high values of the entropy measure (a standardized index based on all posterior probabilities varying between 0 and 1) (38) are preferred. The third criterion concerns the likelihood ratio tests; in our case, we used the Bootstrap Likelihood Ratio Test (BLRT). Non-significant *p*-values (>0.05) of the *k*+1 class solution means that the significant *k*-solution (with *p* < 0.05) is superior. The fourth criterion for a *k*-class solution is based on practical and theoretical considerations (39). After deciding on a *k*-class solution, we labeled the classes based on the patients' HoNOSCA response patterns. The patients were allocated to one of the *k* classes based on their highest subgroup posterior probability (probability of class membership for each patient).

#### Follow-Up Analyses With SPSS

We then tested whether the identified subgroups differed significantly on clinical characteristics and psychosocial outcomes using ANOVA for interval variables and Fisher's exact test for the nominal variables, followed by Bonferroni corrected *post-hoc* tests to test which classes differed from each other. To correct class uncertainty, the individual posterior probabilities were used as a weight variable in additional analyses.

## Results

### Baseline Characteristics of the Youth Flexible ACT Sample

The monitoring sample (*n* = 199) comprised 101 girls (50.8%) and 98 boys (49.2%) with a mean age of 18.6 years (*SD* = 2.49; range 12–24 years of age). Most patients were between 15 and 22 years of age (87.9%), and a large group of the patients was 18 years of age or older (69.3%; range 12–24 years of age). The participants were mainly born in the Netherlands (95%). On average, teams indicated that 17.0% of the caseloads had a parent of foreign origin (non-Dutch). The most frequent clinical diagnoses (Table 2) were trauma and stressor-related disorders (27.1%; of which 80.9% were posttraumatic stress disorders), mood disorders (27.1%), autism spectrum disorder (26.1%), anxiety disorders (23.6%), and attention deficit hyperactivity disorder (ADHD; 21.6%). Most patients had two or more clinical disorders (62.8%) and faced additional problems (62.3%), the so-called V-code or “other conditions that might be a focus of clinical attention” in Diagnostic and Statistical Manual of Mental Disorders (DSM5) (40).

**Table 2:** Clinical diagnosis and care characteristics of the monitoring sample (*n* = 199).

	**Monitoring sample (*n* = 199)**
	***n*** **(%)**
**Clinical diagnosis**	
Trauma and stressor related disorders	54 (27.1)
Mood disorders	54 (27.1)
Autism spectrum disorder	52 (26.1)
Anxiety disorders	47 (23.6)
Attention-deficit/hyperactivity disorder	43 (21.6)
Personality disorders	31 (15.6)
Disruptive, impulse-control and conduct disorders	16 (8.0)
Substance use disorders	15 (7.5)
Psychotic disorders	7 (3.5)
Missing	8 (4.0)
**Referral**	
Specialized mental health care	91 (45.8)
Department inside same mental	
health care institute	64 (70.3)
General practitioner	28 (14.1)
Other (mental) health care institute	27 (13.6)
District social service teams	22 (11.1)
Parents / patient	13 (6.5)
After crisis assessment	10 (5.0)
School	6 (3.0)
Missing	0 (0.0)
**Previously received care** ** <6 months before Youth Flexible**	
**ACT care:**	
Specialized mental health care	93 (46.3)
District social service teams	52 (26.9)
Psychologist center	64 (31.7)
Youth care	42 (22.6)
Other health care institute	40 (18.1)
Medical health care specialist	66 (18.1)
No previous care	16 (8.0)
Missing	4 (2.0)

A large group of patients was referred by a specialized mental health care organization (45.8%), mostly within the same organization that deployed the Youth Flexible ACT team (70.3%). Accordingly, most patients received care from a specialized mental health care organization (46.3%) in the 6 months preceding the Youth Flexible ACT care. A small percentage (8%) did not receive care within the 6 months before the Youth Flexible ACT care. A few patients (8%) already received Youth Flexible ACT care in the past. In 11.6% of the cases, participants had a family member who also received Flexible ACT care.

### Subgroups Within the Youth Flexible ACT Sample

**Figure 1:**
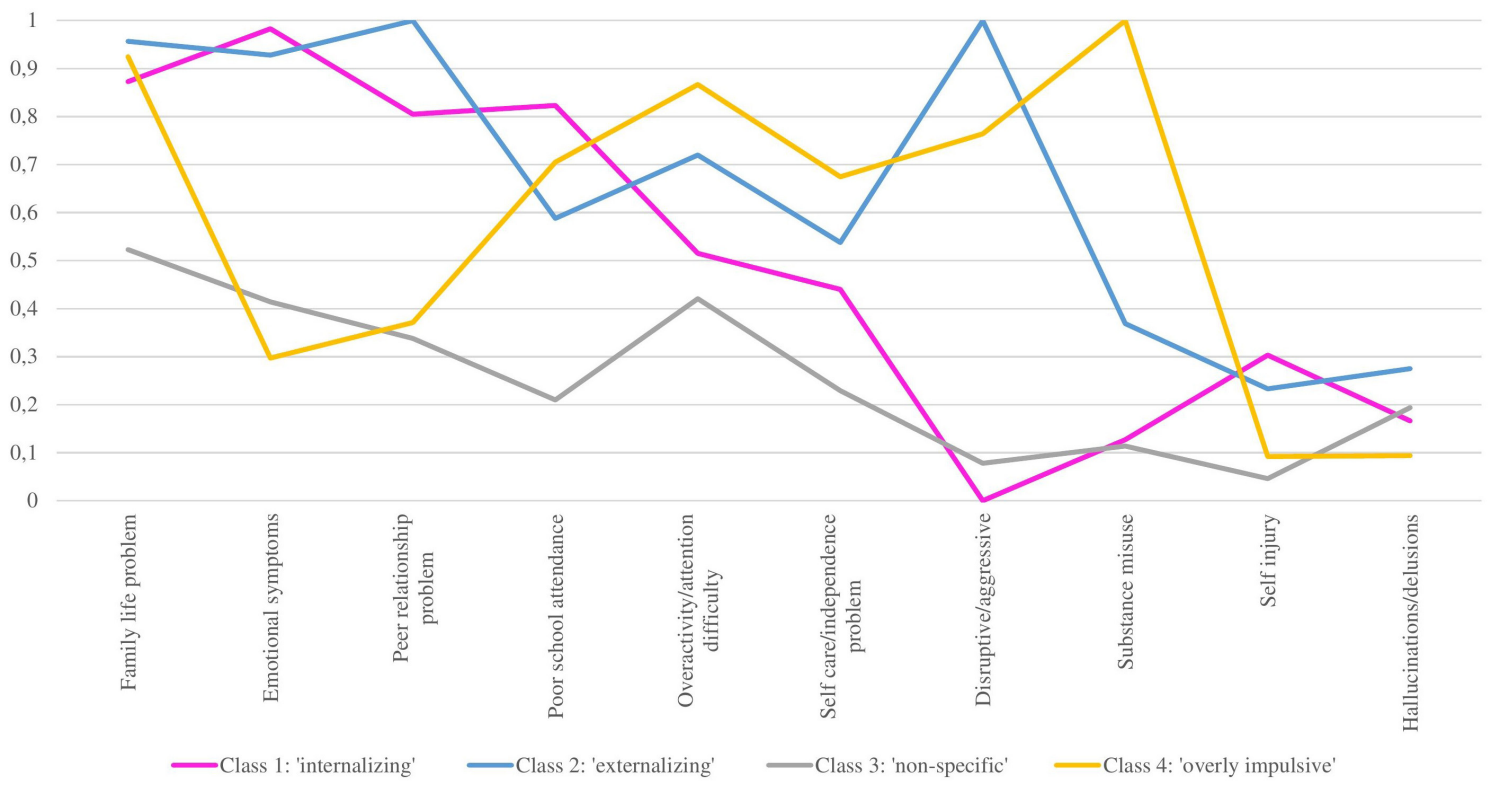
Probability of having a mild/severe problem per class on 10 HoNOSCA items.

We aimed to explore the existence of underlying subgroups within the overall Youth Flexible ACT population. LCA revealed four subgroups of patients (*n* = 199; Table 3). The SSABIC-values decreased from 2,313 to 2,239 for a 4-class solution and then increased again for a 5-class solution, indicating that a 4-class solution is preferred. The entropy-values of the 3-, 4, and 5- class solutions did not show large differences (0.86 −0.88), and the posterior probabilities were high (>0.90), except for class 4 in the 4-class solution and classes 4 and 5 in the 5-class solution. Deciding on a specific class-solution based on entropy and posterior probabilities was difficult. The Bootstrap Likelihood Ratio Test (BLRT) showed a *p*-value of 0.113 for the 4-class solution and a *p*-value of 0.500 for the 5-class solution, indicating a 3-class solution is preferred for statistical reasons. The decision between a 3-class (BLRT) and a 4-class solution (SSABIC) is, therefore, rather arbitrary. The first three classes of the 4-class solution are the same as the three classes of the 3-class solution. We, therefore, first described these classes (Figure 1) before adding a fourth class. The first class (*n* = 87) had a high prevalence (>80%) of emotional symptoms, family life problems, peer relationship problems, and poor school attendance. The second class (*n* = 65) had a high prevalence of disruptive/aggressive behavior, peer relationship problems, emotional symptoms, and family life problems. The most important difference between class 1 and class 2 concerned disruptive/aggressive behavior, with a prevalence of 0% in class 1 and 100% in class 2. We labeled class 1 as the “internalizing” subgroup and class 2 as the “externalizing” subgroup. The third class, labeled as the “non-specific” subgroup (*n* = 32), had a prevalence of 4.6–52.3% across the 10 items and had a relatively high prevalence of family life problems, overactivity/attention difficulties, emotional symptoms, and peer relationship problems. The fourth class, labeled as “overly impulsive” (*n* = 15), had a high prevalence of family life problems, overactivity/attention difficulties, and disruptive/aggressive behavior. All members of this class scored maximally on substance misuse. This latter behavior sets this class apart from the first three. We, therefore, opted for the 4-class solution.

**Table 3:** Latent class models with up to 5 classes.

**LCA**	**Classes**	***N***	**Posterior probability**	**BLRT*p*-value**
Class = 1	1	199	1.000	
SSABIC = 2,313				
Classes = 2	1	162	0.945	0.000
SSABIC = 2,270	2	37	0.859	
Entropy = 0.741				
Classes = 3	1	88	0.933	0.000
SSABIC = 2,243	2	77	0.988	
Entropy = 0.877	3	34	0.914	
Classes = 4	1	87	0.926	0.113
SSABIC = 2,239	2	65	0.965	
Entropy = 0.858	3	32	0.917	
	4	15	0.841	
Classes = 5	1	88	0.932	0.500
SSABIC = 2,243	2	44	0.970	
Entropy = 0.879	3	35	0.910	
	4	17	0.844	
	5	15	0.879	

*BLRT, Bootstrapped likelihood ratio test; SSABIC, Sample Size Adjusted BIC*.

#### Differences Between Subgroups

We next examined differences between the classes in age, sex, and clinical diagnoses. Statistical results are described in Table 4, and any differences described in this paragraph were all significant at *p* < 0.05 after Bonferroni correction. A one way ANOVA indicated no significant differences in age between the four classes, *F*_(3, 181)_ = 0.62, *p* = 0.602. For the other (nominal) variables, Fisher's exact test was used. *Post-hoc* tests with Bonferroni correction were applied only if the *p*-value of Fisher's exact test was significant. Latent class membership was associated with sex and the prevalence of anxiety and mood disorders, disruptive, impulse-control, and conduct disorders, ADHD, and psychotic disorders. The “internalizing” subgroup consisted mostly of girls (64.4%) who had to deal primarily with anxiety and mood disorders (63.2%). The “overly impulsive” subgroup had a higher prevalence of ADHD (53.3%) compared to the “internalizing” subgroup (13.8%) and the “non-specific” subgroup (15.6%) and a higher prevalence of conduct disorders (26.7%) compared to the “internalizing” subgroup (3.4%). In contrast to the other subgroups, the “non-specific” subgroup was characterized by a more or less even pattern of problems across all domains without clear highlights. This subgroup had the highest prevalence of psychotic disorders (15.6%) compared to the other groups, although the absolute number was still relatively low.

**Table 4:** Mean age, sex, and clinical diagnoses of the overall sample and per class.

	**Total sample**	**Class 1**	**Class 2**	**Class 3**	**Class 4**	***p*-value**	***Post-hoc***
*n* (%)	199	87 (43.7)	65 (32.6)	32 (16.1)	15 (7.5)		
Age *M* (*SD*)	18.57 (2.49)	18.72 (2.38)	18.21 (2.72)	18.82 (2.31)	18.65 (2.48)	0.602	
Girls (%)	101 (50.8)	56 (64.4)	28 (43.1)	12 (37.5)	5 (33.3)	0.003	1 > 3
Anxiety & Mood disorders (%)	90 (45.2)	55 (63.2)	19 (29.2)	14 (43.8)	2 (13.3)	0.000	1 > 2 1 > 4
Trauma and Stressor related disorders (%)	54 (27.1)	23 (26.4)	18 (27.7)	9 (28.1)	4 (26.7)	0.993	
Disruptive, Impulse-control, and Conduct disorders (%)	16 (8.0)	3 (3.4)	8 (12.3)	1 (3.1)	4 (26.7)	0.027	4 > 1
Autism spectrum disorder(%)	51 (25.6)	18 (20.7)	19 (29.2)	11 (34.4)	3 (20.0)	0.315	
Attention-deficit/ Hyperactivity disorder (%)	43 (21.6)	12 (13.8)	18 (27.7)	5 (15.6)	8 (53.3)	0.004	4 > 1 4 > 3
Personality disorders (%)	32 (16.1)	17 (19.5)	10 (15.4)	4 (12.5)	1 (6.7)	0.642	
Psychotic disorder (%)	7 (3.5)	1 (1.1)	1 (1.5)	5 (15.6)	0 (0)	0.005	3 > 1 3 > 2

### Life Domains Outcomes for the Youth Flexible ACT Sample and Its Subgroups

To provide a thorough overview of the Youth Flexible ACT sample and to further clarify its subgroups, we measured a broad range of psychosocial and daily functioning outcomes. We allocated these outcomes into the psychological functioning, daily functioning, and intellectual functioning domains. Below, we first provide the descriptives of the total population for each theme and next describe differences between the classes. Numerical results (descriptives and statistical results) are presented in Table 5 (patient-reported), Table 6 (parent-reported), and Table 7 (clinician-reported). In Table 5, interval variables were tested with one-way ANOVA and nominal variables with Fisher's exact test. Both tests were followed by *post-hoc* tests with Bonferroni correction if the main results are significant. All differences described in the text below were significant at *p* < 0.05 after Bonferroni correction. As we did not gather enough responses from parents/carers for each subgroup, additional analyses to compare the classes on parent-reported outcomes could not be performed.

**Table 5:** Descriptives of patient-reported outcomes.

	**Total sample *M* (*SD*)**	**Total sample Range**	**Total sample Frequencies**	**Class 1 *M* (*SD*)**	**Class 2 *M* (*SD*)**	**Class 3 *M* (*SD*)**	**Class 4 *M* (*SD*)**	***F***	***p*-value**	***Post-hoc***
SDQ	*n* = 198			*n* = 87	*n* = 65	*n* = 31	*n* = 15			
Emotional symptoms	5.3 (2.99)	0–10		6.6 (2.66)	4.9 (2.65)	3.9 (3.10)	2.5 (2.30)	F_(3, 180)_ = 16.08	0.000	1 > 2,3,4
Conduct problems	2.6 (1.93)	0–8		1.9 (1.65)	3.2 (1.84)	3.0 (2.34)	3.4 (1.60)	F_(3, 180)_ = 7.50	0.000	2 > 4 2,4 > 1
Hyperactivity- inattention problems	5.3 (2.56)	0–10		5.4 (2.64)	5.4 (2.51)	4.7 (2.63)	5.6 (2.10)	F_(3, 180)_ = 0.89	0.446	n.a.
Peer relationship problems	3.1 (1.94)	0–9		3.3 (2.08)	3.2 (2.06)	2.8 (1.41)	2.1 (1.1)	F_(3, 180)_ = 2.00	0.116	n.a.
Total score	16.3 (6.16)	1–31		17.2 (5.92)	16.6 (5.95)	14.3 (7.50)	13.5 (3.60)	F_(3, 180)_ = 2.95	0.034	n.a.
Cut-off score ≥ 16			112 (56.6%)	54 (62.1%)	42 (64.6%)	11 (35.5%)	5 (33.3%)		0.007	2 > 3
Impact score (*n* = 169)	3.7 (2.72)	0–10		4.5 (2.75)	3.4 (2.63)	2.6 (2.46)	2.1 (1.85)	F_(3, 153)_ = 6.17	0.001	1 > 3,4
Cut-off score ≥ 2			125 (74.0%)	63 (82.9%)	41 (70.7%)	13 (59.1%)	8 (61.5%)		0.036	n.a
CDI-2	*n* = 199			*n* = 87	*n* = 65	*n* = 32	*n* = 15			
Total score	18.9 (10.14)	0–47		22.3 (10.68)	17.4 (9.25)	15.5 (8.55)	12.9 (7.24)	F_(3, 181)_ = 7.56	0.000	1 > 2,3,4
Cut-off score ≥ 14			129 (64.8%)	65 (74.7%)	40 (61.5%)	18 (56.3%)	6 (40.0%)		0.025	1 > 4
PQ-16	*n* = 197			*n* = 86	*n* = 65	*n* = 31	*n* = 15			
Total score	5.6 (3.78)	0–15		5.9 (3.63)	6.0 (4.10)	4.9 (3.84)	4.0 (2.70)	F_(3, 179)_ = 1.63	0.184	n.a
Cut-off score ≥ 6			97 (49.2%)	45 (52.3%)	33 (50.8%)	13 (41.9%)	6 (40.0%)		0.646	n.a.
Distress score (*n* = 183)	8.7 (7.06)	0–36		9.2 (7.43)	9.5 (6.81)	7.4 (7.28)	5.2 (3.63)	F_(3, 166)_ = 1.91	0.130	n.a.
Social support and peers	*n* = 198			*n* = 86	*n* = 65	*n* = 32	*n* = 15			
T-score	63.5 (12.92)	30–92		60.9 (13.10)	63.3 (12.91)	66.5 (11.54)	73.4 (9.15)	F_(3, 180)_ = 5.08	0.002	4 > 1,2
Cut-off score <40			12 (6.1%)	6 (7.0%)	5 (7.7%)	1 (3.1%)	0 (0.0%)		0.825	n.a.
Satisfaction with peer relationships	6.8 (2.42)	1–10	149 (75.3%)	6.19 (2.54)	7.03 (2.28)	7.53 (2.17)	8.33 (1.68)	F_(3, 180)_ = 5.61	0.001	3,4 > 1
Score ≥ 6				59 (68.6%)	50 (76.9%)	26 (81.3%)	14 (93.3%)		0.158	n.a.
Kidscreen-10	*n* = 199			*n* = 87	*n* = 65	*n* = 32	*n* = 15			
Total score (T-score)	40.1 (4.60)	25–57		39.0 (4.28)	40.5 (4.88)	41.4 (3.91)	42.1 (5.28)	F_(3, 181)_ = 3.59	0.015	n.a.
Cut-off score <40			95 (47.7%)	51 (58.6%)	28 (43.1%)	10 (31.3%)	6 (40.0%)		0.042	n.a.
Empowerment	*n* = 198			*n* = 86	*n* = 65	*n* = 32	*n* = 15			
Total score (T-score)	46.9 (11.32)	23–80		45.3 (11.83)	47.7 (9.97)	50.3 (11.67)	46.1 (12.32)	F_(3.180)_ = 1.81	0.147	n.a.
Cut-off score <35			40 (20.2%)	23 (26.7%)	9 (13.8%)	4 (12.5%)	4 (26.7%)		0.168	n.a.
Satisfaction with:	*n = 199*			*n = 87*	*n = 65*	*n = 32*	*n = 15*			
-School/employment	5.52 (2.36)	1–10		5.15 (2.36)	5.40 (2.40)	6.31 (2.25)	6.53 (1.89)	F_(3, 181)_ = 3.05	0.030	n.a.
Score ≥ 6			116 (58.3%)	42 (48.3%)	40 (61.5%)	23 (71.9%)	11 (73.3%)		0.041	n.a.
-Leisure time	6.31 (2.36)	1–10		5.90 (2.45)	6.38 (2.42)	6.91 (2.09)	7.13 (1.60)	F_(3, 181)_ = 2.31	0.078	n.a.
Score ≥ 6			129 (64.8%)	44 (50.6%)	45 (69.2%)	27 (84.4%)	13 (86.7%)		0.001	3 > 1
-Housing	6.56 (2.48)	1–10		7.06 (2.35)	5.88 (2.75)	6.75 (2.10)	6.27 (2.22)	F_(3, 181)_ = 2.89	0.037	1 > 2
Score ≥ 6			139 (69.8%)	68 (78.2%)	38 (58.5%)	23 (71.9%)	10 (66.7%)		0.082	n.a.
Personal finance	*n = 199*			*n = 87*	*n = 65*	*n = 32*	*n = 15*			
Having financial problems			43 (21.6%)	20 (22.9%)	13 (20.0%)	6 (18.8%)	4 (26.7%)		0.202	n.a.
The legal system and police	*n = 199*			*n = 87*	*n = 65*	*n = 32*	*n = 15*			
Contact with police			46 (23.1%)	12 (13.8%)	23 (35.4%)	4 (12.5%)	7 (46.7%)		0.000	2,4 > 1

**Table 6:** Descriptives of parent-reported outcomes.

	**Total sample *M* (*SD*)**	**Total sample range**	**Total sample frequencies**	**Class 1 *M* (*SD*)**	**Class 2 *M* (*SD*)**	**Class 3 *M* (*SD*)**	**Class 4 *M* (*SD*)**
SDQ-P	*n* = 71			*n* = 35	*n* = 25	*n* = 9	*n* = 2
Emotional symptoms	6.0 (2.43)	1–10		6.3 (2.24)	6.2 (2.57)	4.4 (2.19)	3.0 (1.41)
Conduct problems	2.7 (2.31)	0–9		2.2 (2.40)	3.7 (1.99)	2.2 (2.39)	2.5 (0.71)
Hyperactivity- inattention problems	5.4 (2.57)	0–10		4.8 (2.56)	6.3 (2.45)	5.6 (2.19)	4.5 (4.95)
Peer relationship problems	3.4 (2.02)	0–7		3.4 (1.97)	3.7 (2.06)	3.0 (2.27)	3.0 (2.83)
Total score	17.5 (6.23)	5–31		16.7 (5.83)	19.9 (6.64)	15.2 (5.43)	13.0 (4.24)
Cut-off score ≥ 14			49 (69.0%)				
Impact score	4.5 (2.97)	0–10		4.7 (2.96)	5.2 (2.99)	2.9 (2.67)	2.6 (0.80)
Cut-off score ≥ 2			56 (78.9%)				
Kidscreen-10 parent report	*n* = 71			*n* = 35	*n* = 25	*n* = 9	*n* = 2
T-score	39.0 (6.05)	29–60		39.0 (5.62)	39.0 (5.31)	41.1 (8.87)	31.0 (3.47)
Cut-off score <40			41 (57.7%)				
PQS-S	*n* = 70			*n* = 35	*n* = 24	*n* = 9	*n* = 2
Total score	22.6 (6.63)	10–36		22.3 (6.26)	23.6 (6.63)	20.3 (7.70)	27.0 (9.90)
Cut-off score ≥ 22			40 (57.1%)				
MHI-5	*n* = 69			*n* = 35	*n* = 24	*n* = 8	*n* = 2
Total score	63.8 (18.84)	20–100		63.5 (20.92)	63.3 (17.28)	66.5 (17.88)	62.0 (8.49)
Cut-off score ≤ 60			32 (46.4%)				

**Table 7:** Descriptives of the HoNOSCA completed by mental health workers (*n* = 199 patients).

	**No problem *n* (%)**	**Minor problem *n* (%)**	**Mild problem *n* (%)**	**Moderately severe problem *n* (%)**	**Severe to very severe problem *n* (%)**	**Unknown/missing *n* (%)**
1. Problems with disruptive, antisocial, or aggressive behavior	72 (36.2)	49 (24.6)	38 (19.1)	22 (11.1)	18 (9.0)	0 (0.0)
2. Problems with overactivity, attention, or concentration	34 (17.1)	50 (25.1)	51 (25.6)	46 (23.1)	16 (8.0)	2 (1.0)
3. Non-accidental self injury	123 (61.8)	32 (16.1)	18 (9.0)	19 (9.5)	6 (3.0)	1 (0.5)
4. Problems with alcohol, substance/solvent misuse	119 (59.8)	26 (13.1)	21 (10.6)	12 (6)	20 (10.1)	1 (0.5)
5. Problems with scholastic or language skills	72 (36.2)	28 (14.1)	37 (18.6)	44 (22.1)	12 (6.0)	6 (3.0)
6. Physical illness or disability problems	141 (70.9)	24 (12.1)	13 (6.5)	16 (8.0)	5 (2.5)	0 (0.0)
7. Problems associated with hallucinations, delusions, or abnormal perceptions	132 (66.3)	27 (13.6)	21 (10.6)	14 (7.0)	5 (2.5)	0 (0.0)
8. Problems with non-organic somatic symptoms	114 (57.3)	39 (19.6)	17 (8.5)	22 (11.1)	7 (3.5)	0 (0.0)
9. Problems with emotional and related symptoms	19 (9.5)	19 (9.5)	34 (17.1)	92 (46.2)	35 (17.6)	0 (0.0)
10. Problems with peer relationships	25 (12.6)	25 (12.6)	43 (21.6)	87 (43.7)	19 (9.5)	0 (0.0)
11. Problems with self-care and independence	65 (32.7)	44 (22.1)	59 (29.6)	21 (10.6)	9 (4.5)	1 (0.5)
12. Problems with family life and relationships	15 (7.5)	17 (8.5)	45 (22.6)	79 (39.7)	42 (21.1)	1 (0.5)
13. Poor school attendance	58 (29.1)	14 (7.0)	16 (8.0)	30 (15.1)	73 (36.7)	8 (4.0)
Total score						
*M* (*SD*) range	19.25 (7.29) 0–42					

#### Psychological Functioning

##### Patient-reported

The average total scores of the patient-reported mental health questionnaires SDQ (psychosocial well-being) (41, 42), CDI-2 (depressive symptoms) (43, 44), and PQ-16 (psychosis risk screening) (45, 46) were substantially higher compared to norm scores of the general population [SDQ: (42, 47–49); CDI-2: (43, 50); PQ-16: (51)] and comparable to the scores of patients in a clinical setting [SDQ: (47, 49, 52, 53); CDI-2: (43, 50, 54); PQ-16 (45, 46, 55)].

Most of the “internalizing” subgroup scored in the clinical range of depression (74.7%; CDI-2 ≥14) and had a significantly higher average total depression score (CDI-2) and emotional difficulties score (emotional problems subscale; SDQ) compared to the other subgroups. The “internalizing” subgroup showed more emotional problems, whereas the “externalizing” and “overly impulsive” subgroups showed more conduct problems (conduct problems subscale; SDQ). On average, all subgroups indicated that their psychosocial problems interfere with their everyday life; however, this problem was more severe for the “internalizing” subgroup compared to the “non-specific” and “overly impulsive” subgroups (SDQ impact score).

##### Parent-reported

The average total score on the parent-reported SDQ (SDQ-P) (41) was comparable to the scores of clinical samples (49, 52, 56). Most parents (69.0%) observed an increased level of psychosocial problems in their child (cutoff ≥14).

##### Clinician-reported

Regarding the HoNOSCA scores, in many cases (80.9%), mental health workers reported the item “Problems with emotional and related symptoms” as “problematic” (mild problem to very severe problem). In more than half of the cases (56.7%), the item “Problems with overactivity, attention, or concentration” was scored as “problematic” while the item “Problems with disruptive, antisocial, or aggressive behavior” was “problematic” in fewer than half of the cases (39.2%). “Problems with alcohol, substance/solvent misuse,” “Non-accidental self-injury,” and “Problems associated with hallucinations, delusions, or abnormal perceptions” were less frequently reported but still evident (respectively 26.7, 21.5, and 20.1%). HoNOSCA scores per class were described previously. They are displayed in Figure 1.

#### Daily Functioning

##### Daytime activities

In summary, 34.7% of the adolescents in the monitoring sample reported going to school, 19.1% reported a combination of school and work, and 12.6% were employed. Nearly all of the adolescents who went to school were in secondary education (94.4%), with most of them (69.3%) in (pre)vocational education. However, about a third of the adolescents (33.7%) did not have school or employment, and more than half (59.7%) of these adolescents did not have any form of organized daily activities. Just above half of the monitoring sample (58.3%) rated their satisfaction with school and/or employment situation higher than six out of 10. In addition, mental health workers indicated problems with school attendance in 62.3% of the cases, as scored by the HoNOSCA. Besides the school and/or employment situation, most participants (64.8%) rated their satisfaction with leisure time higher than six out of 10. The “non-specific” subgroup was more satisfied with leisure time compared to the “internalizing” subgroup (84.4 vs. 50.6%).

##### Family life and housing situation

Regarding their housing situation, most adolescents were living with both their biological parents (34.2%), with one biological parent (28.1%), or with blended families (5.0%). Almost one-fifth of the adolescents (18.6%) were in assisted living. Others lived on their own, with friends, or had another form of housing situation. Mental health workers indicated that, on average, a small percentage of their caseload (8.0%) lived in a residential facility (including psychiatric hospitalization). Adolescents themselves were slightly satisfied with their housing situation, with 69.8% scoring above 6 out of 10. The “internalizing” subgroup was more satisfied with their housing situation compared to the “externalizing” subgroup (78.2 vs. 58.5%). Although adolescents were quite satisfied with their housing situation, mental health workers noticed “Problems with family life and relationships” in most cases (83.4%; HoNOSCA). In addition, a large group of parents (57.1%) experienced a considerable degree of parenting stress (cut-off ≥22; PSQ-S) (57, 58). The reported parenting stress total score was substantially higher compared to norm scores for a non-clinical sample (57) and comparable with scores for a clinical sample (59). Moreover, almost half of the parents (46.4%) reported experiencing psychological distress according to Dutch norms (cutoff ≤60; MHI-5) (60).

##### Peer relationships

On average, adolescents reported experiencing enough quality of social interaction with peers [Social Support and Peers' subscale of the Kidscreen-52; (61)] compared to the norm scores from both the general population and clinical samples (62, 63). Quite a large group (75.3%) scored above six out of 10 and indicated satisfaction with their peer relationships. Although on average, each subgroup experienced enough quality of interaction with their peers, the “overly impulsive” and “non-specific” subgroups were more satisfied compared to the “internalizing” and “externalizing” subgroups. A major contrast to these results is that mental health workers noted “Problems with peer relationships” of the HoNOSCA as “problematic” in 74.8% of the cases.

##### Personal finance

One-fifth of the total sample (21.6%) reported having debts or financial problems in the past 6 months. This percentage is similar to that for the subgroups.

##### Legal system and police

Almost a fourth of the adolescents (23.1%) reported having been in contact with the legal system/police in the past 6 months. The “externalizing” and “overly impulsive” subgroups (respectively 35.4 and 46.7%) reported having been more frequently in contact with the legal system/police compared to the “internalizing” subgroup (13.8%). Mental health workers reported that only a small number of their caseload (0.6%) was in juvenile detention.

##### Health-related quality of life

On average, adolescents reported satisfactory quality of life on the KIDSCREEN-10 according to European norms (61, 63). However, when examining the frequencies, almost half of the adolescents (47.7%) indicated experiencing poor quality of life (cut-off <40). Additionally, more than half of the parents (57.7%) indicated that their child had a poor quality of life (cut-off <40) (61).

##### Empowerment

Many adolescents reported having control over decisions and actions (79.8%), as scored on the EMPO 3.1 (58, 64). The remaining 20.2% indicated that empowerment needs to be a subject of attention during the treatment (cut-off <35). No significant difference in the quality of life and empowerment were observed between classes.

#### Intellectual Functioning

Intellectual functioning can be divided into Borderline Intellectual Functioning (IQ 70–85) and Mild Intellectual Disability (IQ 50–69) (40). Intelligence Quotient (IQ) scores were collected in 41.7% of the cases (21.6% <2 years; 20.1% >2 years ago; 20.6% missing scores). The collected scores showed an average IQ score of 92.6 (*SD* = 17.89). About one third (33.8%) had an IQ score below 85. Mental health workers indicated that ~18.6% of the total sample had to deal with (suspected) below average intellectual functioning. No significant differences between the classes were observed.

## Discussion

In this paper, we aimed to characterize adolescents in Youth Flexible ACT care. We presented the baseline findings of our prospective cohort study, including sociodemographic-, clinical characteristics, and psychosocial outcomes. To our knowledge, this is the first characterization of the Youth Flexible ACT target group.

The Youth Flexible ACT target group consisted mainly of adolescents aged 15 and 22 who already received prior (specialized) mental healthcare. As expected, they face many severe psychiatric and social problems associated with a significant amount of trauma and developmental, mood, and anxiety disorders. Their development in multiple life domains is hindered, especially since one third does not attend a school or a job, and almost all adolescents show problems with family life and peer relationships. About half reported experiencing poor quality of life. Other frequently reported difficulties are substance misuse, the involvement of the legal system or police, problems with intellectual functioning, and personal finance. On a positive note, most adolescents indicated feeling empowered: the feeling that they can control their decisions and actions, which affect their health (58).

Our results showed that the Youth Flexible ACT target group could be divided into four subgroups: the (1) “internalizing,” (2) “externalizing,” (3) “non-specific,” and (4) the “overly impulsive” subgroup. The “internalizing,” “externalizing,” and “overly impulsive” subgroups showed severe problems in multiple life domains, each with its unique difficulties. The “non-specific” subgroup displayed a milder and diffuse pattern of problems across all domains without clear highlights. This subgroup had the highest percentage of psychotic disorders and autism spectrum disorders. The existence of this generic subgroup could be explained by problems remaining under the radar for some adolescents at the start of Youth Flexible ACT. While some adolescents apply for Youth Flexible ACT on their own volition, other adolescents are registered by other concerned parties, such as parents, caregivers, educators, health care professionals, or law enforcement. In these latter cases, adolescents probably may not (yet) recognize or acknowledge the problems they encounter, which can (temporarily) result in “meddling care” (intervention without the clear consent of the person). In these cases, time is initially devoted to building and maintaining trust and motivation, while problem diagnostic is secondary. Another explanation for this generic subgroup comprising a relatively large number of patients with mild psychotic symptoms could be the presence of “early intervention in psychosis” teams (65) in the region. These teams work with young adults who deal with psychosis. In this case, young adults with somewhat milder psychotic symptoms will then get treatment and support from Youth Flexible ACT teams or traditional outpatient care.

Since each subgroup has its own set of difficulties, appropriate care should be embedded in the Youth Flexible ACT team care services. For example, expert knowledge about developmental disorders and psychosis must be available in the teams. Additionally, family and pedagogical interventions and addiction interventions are highly needed. Considering the disruptive behavior and impulse control problems, interventions targeting self-control, aggression regulation, social and problem-solving skills are relevant. Although these services are already mentioned in the Youth Flexible ACT model description (7), our findings reiterate the importance of their presence in the teams. Taken together, our findings underscore the need for an integrated care approach. These teams should consist of a team of multidisciplinary professionals who can address the multiple needs across psychiatric and social services. For future research, it would be interesting to explore different improvement trajectories of these subgroups through longitudinal tracking of psychosocial outcomes.

### Findings in Perspective

In general, an integrated care approach to treating adolescents with persistent complex care needs is vital. Although the Youth Flexible ACT model is prominent in the Netherlands (27), other youth-friendly mental health and integrated services are on the rise in other countries (14, 66–68). International examples include IMYOS (Intensive Mobile Youth Outreach Service) in Australia, Jigsaw in Ireland, Forward Thinking Birmingham in the United Kingdom, and ACCESS Open Minds in Canada (14, 21, 24, 68, 69). The patient group of the IMYOS is similar (e.g., poor school attendance, problematic upbringing, history of mainstream mental health services before referral) yet slightly more focused on “high risk” youth compared to the Youth Flexible ACT patients (70, 71). In contrast to the Youth Flexible ACT approach, most other community-based centers provide (mental health) services for youth in the primary care setting or focus specifically on particular diagnoses, often psychotic disorders. The Youth Flexible ACT model is unique because it encompasses a multi-agency care approach delivering long-term assertive outreach care for wide-ranging problems. Multidisciplinary teams provide psychiatric interventions in a specialized mental health care setting in close collaboration with adolescents (and their families) and professionals from other youth services, thereby delivering a range of services while maintaining continuity of care. In subsequent (inter)national studies, it would be interesting: (1) to see how the comparable models and target groups relate to each other and (2) to establish the benefits and relevance of integrated care.

In addition, most patients of our research sample were between 15 and 22 years of age, also referred to as transitional age youth. Continuity of care in transitional age youth has a high priority in the global mental healthcare. In the Netherlands, the Flexible ACT model for adults is an established model, whereas the youth version is a more recent development. Our findings show that the Youth Flexible ACT caseload is substantially different compared to the Adult Flexible ACT patient group, which predominantly focuses on treating severe and enduring mental disorders, such as psychotic-, personality-, and substance use disorders (72–74). The transition of 18+ Youth Flexible ACT patients to Adult Mental Health Services (AMHS) is complicated because of the variable organization of service provision and the limited access to the required services in AMHS (67, 68, 75, 76). By incorporating a broad age group, Youth Flexible ACT attempts to prevent transitional age youth from falling through the gap between child and adolescent mental health services and AMHS. In addition, as the Youth Flexible ACT model is composed of multiple professionals and areas of expertise, it addresses the age-related concerns of both young children and young adults.

Furthermore, we note that 17% of youths had at least one parent of foreign origin. This is slightly lower than the Dutch national average of 27% (77). This could be due to the fact that this percentage is generally lower in rural areas, and that our teams are predominantly located in rural areas. Alternatively, it might be possible that youths with a migration background have additional difficulty in navigating the Dutch mental health care system, or potentially seek less help due to cultural differences, in turn leading to underrepresentation in the specialized mental health care settings and thus Youth Flexible ACT care. However, there are no current national figures that outlines the number of children and adolescents from either migrant or Dutch origin in specialized youth mental health care. Yet, the percentage of migrants in the Youth Flexible ACT sample is comparable with that of other studies with young patients from clinical populations in the Netherlands (46, 50, 54).

### Strengths and Limitations

An important strength of the current study is its observational and naturalistic character, which improves the external validity because the data closely represents everyday practice. The study examined Youth Flexible ACT in daily practice across multiple Youth Flexible ACT teams and mental health care centers throughout the Netherlands. In addition, the study included a relatively large study sample representative of the Youth Flexible ACT population in the Netherlands, which is a difficult patient group to study. Furthermore, using both a broad set of questionnaires (both psychiatric and social functioning) and data from multiple informants, this study provides a comprehensive view from multiple vantage points. This in-depth framework can be used by other researchers to perform comparisons.

Several limitations of our study should be noted as well. First, mental health workers were not always able to obtain a complete overview of the problems for some patients due to the difficult-to-engage nature of the patient group. This can lead to underestimation of diagnoses and problems. Second, the downside of including a broad age group is that the employed questionnaires have not been extensively validated with older adolescents. This should be considered when interpreting the results. Third, our results showed that problems related to psychosocial well-being and peer relationships were reported more often by clinicians and parents than by patients themselves. This is in line with other observations that parents and mental health workers generally identify more problems than the patients themselves (47, 52, 78–80). Finally, although the study population was sufficiently large to perform LCA analysis, it should be noted that the individual subgroup sizes were sometimes relatively small (minimally *n* = 15). Finding statistically significant differences in small sample sizes is only possible if the differences have large effect sizes. As we found multiple statistically significant effects (with sufficient power to describe our study population), this indicates that our effect sizes were large. For finding significant differences with medium or small effect sizes some subgroups were too small indicating that replication of these results with larger samples is recommended before policies regarding targeted and specialized care toward one of the subgroups is to be set in place.

In conclusion, the multitude of psychiatric and social problems found in the current study highlights the vulnerability of the population requiring specialized forms of care that can bridge the wide-ranging problems. We await the results of our longitudinal study that will show whether Youth Flexible ACT is indeed successful in engaging and helping adolescents with persistent complex problems.

## Data Availability Statement

The datasets generated for this article are not readily available because the datasets analyzed for this study will not be made publicly available due to ethical, legal, and privacy restrictions. Requests to access the datasets should be directed to m.broersen@ggzoostbrabant.nl.

## Ethics Statement

The study involving human participants was reviewed and approved by the Trimbos Ethics Committee. Written informed consent to participate in this study was provided by adolescents and parents or legal guardians.

## Author Contributions

All authors contributed to the article and approved the submitted version. MB was responsible for data collection, data management, and reporting the study results. MB and AV were responsible for conducting and reporting data analysis. NF, DC, and HK read the manuscript and provided suggestions for improvement. NF, DC, and HK also served as supervisors.

## Conflict of Interest

HK was a board member of the Centre for Certification ACT and Flexible ACT (CCAF). The CCAF executed peer audits using model fidelity scales to ensure the quality of ACT and Flexible ACT. The remaining authors declare that the research was conducted in the absence of any commercial or financial relationships that could be construed as a potential conflict of interest.

## Abbreviations

ACT: Assertive Community Treatment
ADHD: Attention Deficit Hyperactivity Disorder
AIC: Akaike Information Criterion
AMHS: Adult Mental Health Services
ANOVA: Analysis of variance
BIC: Bayesian Information Criterion
BLRT: Bootstrap Likelihood Ratio Test
CCAF: Centre for Certification ACT and Flexible ACT
CDI-2: Child Depression Inventory 2
DSM: Diagnostic and Statistical Manual of Mental Disorders
EMPO 3.1: Empowerment questionnaire 3.1
HoNOSCA: Health of the National Outcome Scales for Children and Adolescents
IMYOS: Intensive Mobile Youth Outreach Service
IQ: Intelligence Quotient
LCA: Latent Class Analysis
MHI-5: Mental Health Inventory 5
PSQ-S: Parenting Stress Questionnaire – short form
PQ-16: The Prodromal Questionnaire 16
SDQ: The Strengths and Difficulties Questionnaire
SDQ-P: Strengths and Difficulties Questionnaire – Parent version
SPSS: Statistical Package for the Social Sciences
SSABIC: Sample Size Adjusted BIC
STROBE: Strengthening the reporting of observational studies in epidemiology.
